# Ontogenetic Variations in the Sensory Organ Structure and Morphology on the Cephalic Appendages of *Hermetia illucens* (Diptera: Stratiomyidae) Larvae

**DOI:** 10.3390/insects17040362

**Published:** 2026-03-25

**Authors:** Yongjun Luo, Rencan Yang, Yan Zhang, Bin Zhang, Dawei Sun, Zhiyong Zhao, Zongbo Li

**Affiliations:** 1 Key Laboratory of Forest Disaster Warning and Control in Yunnan Province, Southwest Forestry University, Kunming 650224, China; lyj9909@outlook.com (Y.L.); zhyan0@outlook.com (Y.Z.); 2 Institute of Livestock and Poultry Breeding Environment Control, Yunnan Academy of Animal Science and Veterinary Medicine, Kunming 650224, China; yangrencan233@163.com (R.Y.); binzhang89@163.com (B.Z.); sdw17787273236@163.com (D.S.)

**Keywords:** black soldier fly, sensilla, antennae, maxillary palp, chemoreceptor

## Abstract

The black soldier fly, *Hermetia illucens* (Linnaeus, 1758), is a globally recognized resource insect for waste bioconversion and sustainable resource provision. Understanding its larval sensory system is key to explaining feeding and environmental perception behaviors. This study used scanning and transmission electron microscopy to analyze the types, distribution, ultrastructure, and developmental consistency of sensilla on larval cephalic appendages. Five sensilla types were found on antennae, sensilla basiconica I–II, sensilla twig basiconica I–II, and sensilla campaniformia, each type comprising 2–6 sensilla. Mouthparts harbored ten types, including sensilla twig basiconica III–V, sensilla placodea, sensilla ligulate, sensilla digitiformia, sensilla trichodea, and sensilla chaetica I–III, with 2–9 sensilla per type. All sensilla showed constant numbers and positions throughout larval development. Ten sensillum types with cuticle pores were innervated by 2–6 sensory neurons, primarily suggesting chemoreceptors; these were concentrated at the tips of antennae and maxillary palps. Sensilla digitiformia on the palps possessed a non-porous cuticle and a single sensory cell, indicative of a thermo-/hygroreceptive role. Sensilla chaetica and trichodea, with non-porous cuticles and no dendrites, were mechanosensory. These results reveal the morphofunctional basis of larval sensation, supporting chemoreception studies and optimizing rearing via behavioral modulation.

## Simple Summary

Black soldier fly larvae help turn diverse organic waste into useful products. To better understand how they feed and sense their environment, we studied the tiny sensory organs on their heads across growth stages using electron microscopy. We found 15 types of these sensory structures: five on the antennae and ten on the mouthparts. Their number and position do not change as the larvae grows. Most have small pores and are connected to 2–6 sensory cells, meaning they likely detect chemicals like smells or tastes. These are mostly found at the tips of the antennae and maxillary palps. One unique, finger-like organ on the palp, linked to just one nerve cell, probably senses temperature and humidity. Other simple, solid hairs are likely used for mechanical sensors. These findings offer insight into morphofunctional features and support future studies on chemoreception and farming improvements.

## 1. Introduction

The black soldier fly (BSF), *Hermetia illucens* (Linnaeus, 1758) (Diptera: Stratiomyidae), is native to the southeastern United States but has become a global distribution due to its versatility in waste bioconversion and sustainable resource provision [1,2]. Notably, BSF larvae serve as a high-protein alternative feed for livestock, poultry, and aquaculture, supporting sustainable nutrition without compromising feed efficiency [1,3,4]. Their production aligns with circular economy principles by recovering minerals from waste streams and converting organic residues into valuable biomass, thereby offering substantial economic and environmental benefits [5,6].

BSF larval development exhibits considerable plasticity, influenced by diet and environmental factors such as temperature and humidity [7,8,9]. Larvae typically undergo five to seven instars [10,11], with the complete life cycle spanning approximately 45 days: egg (∼4 days), larva (13–18 days), prepupa (7–14 days), and adult (5–9 days). As development proceeds, larval pigmentation intensifies. Upon reaching the prepupal stage, larvae cease feeding, clear their gut contents, and exhibit reduced motility, while the cuticle hardens and accumulates calcium, forming a darkened protective envelope. During this phase, nutrient metabolism shifts toward the utilization of reserves accumulated earlier, indicating that organic waste conversion occurs predominantly from the third through sixth instars. The prepupal stage represents the optimal harvest point in BSF production. At this juncture, gut clearance minimizes pathogen risk, while the concurrent conversion and concentration of nutrients facilitate efficient biomass collection [12,13].

Currently, large-scale separation of larvae from frass and residual substrate relies primarily on mechanical methods [14,15]. These techniques can be broadly categorized as either active, utilizing stimuli such as oxygen, light, or heat, or passive, including sieving, air classification, and centrifugal separation. However, these existing separation strategies are often hampered by low efficiency, high labor requirements, elevated operational costs, and inconsistent output quality due to mixed larval instars. These limitations underscore the need for a simpler, more efficient separation approach. A promising alternative involves exploiting the innate behavioral responses of larvae and prepupae [16], particularly their positive taxis toward suitable food sources and pupation sites, a process governed by sensory mechanisms [10,17]. Exploiting these directional responses could facilitate the selection of homogeneously developed individuals and enhance overall resource utilization in rearing systems [18,19]. Hence, understanding the larval sensory biology of *H. illucens* is essential for advancing both future research and industrial applications.

Unlike the adult sensory system, which relies heavily on olfaction to locate mates, oviposition sites, and food over long distances [20,21,22,23], larval sensory requirements are more constrained by limited mobility and restricted habitats [22,24,25]. Consequently, larval olfaction is typically limited to short-range orientation toward food sources or detection of aggregation pheromones [26], while gustation and tactile cues play a more dominant role in mediating food selection and responses to mechanical, thermal, and humidity stimuli [24,27,28]. Furthermore, sensilla are shed and renewed with each molt, and their abundance and diversity vary across instars and species, reflecting adaptive specializations shaped by life history, ecological niche, dietary breadth, and developmental stage [21,24,25]. In some cases, species with similar lifestyles or close phylogenetic relationships also exhibit shared or analogous sensillar configurations [29,30,31]. For instance, the polyphagous lepidopteran larvae of *Yponomeuta* species possess six types of cephalic sensilla (e.g., sensilla styloconica, basiconica) primarily involved in detecting host plant volatiles [27]. In contrast, oligophagous wood-boring coleopteran larvae (e.g., Cerambycidae) exhibit distinct groups of digitiform and placoid sensilla on the apical segments of antennae and mouthparts [25], while apodous dipteran larvae (e.g., Cecidomyiidae) display convergent morphologies such as sensilla placodea and microampullacea [32,33]. These variations underscore a direct link between sensilla distribution and feeding ecology [34]. Nevertheless, detailed morphological analyses of the mouthpart and antennal sensilla in larvae of many Stratiomyidae subfamilies, including *H. illucens*, remain lacking [18].

To date, only two studies have described the cephalic sensilla of *H. illucens* larvae. Barros et al. [10] documented elongated sensilla on the second antennal segment across larval instars 1–7, while Fabian et al. [35] briefly noted the presence of sensilla on larval head appendages during their investigation of cephalic structural transformations. More importantly, no study has provided a detailed account of the internal structure of these sensilla, a prerequisite for accurately predicting their sensory functions and for guiding further electrophysiological and molecular analyses.

In this context, the present study uses scanning and transmission electron microscopy to investigate the external and internal morphology of the cephalic sensilla in *H. illucens* larvae. Through detailed examination of the fine structure of the antennae and mouthparts, we aim to infer sensillar functions and characterize ontogenetic and functional morphological variations. These results provide an essential basis for electrophysiological studies, still unexplored in Stratiomyidae larvae, and offer insights that may contribute to optimizing artificial breeding and waste processing systems.

## 2. Materials and Methods

### 2.1. Instar Determination and Sample Collection of BSF Larvae

To obtain larvae of different instars, a cluster of approximately 300–500 eggs was placed on chicken manure substrate under controlled environmental conditions (29 ± 1 °C, 65% relative humidity, 12:12 h (light:dark) photoperiod). Upon hatching, newly emerged individuals were regarded as first-instar larvae. Subsequent instars were determined following the morphological descriptions of Barros et al. [10] and Kim et al. [36], using cephalic width as the primary criterion, supplemented by body coloration and developmental duration (Table 1; Figure S1). Individuals reared beyond 18 days that exhibited an elongated, dorso-ventrally flattened body with a ridged, rigid cuticle retaining the mosaic pattern were classified as seventh-instar or prepupal stage.

For specimen preparation, 20 larvae per instar were collected, rinsed with 0.1 mol/L Ringer’s solution (0.85% NaCl, 0.03% KCl, 0.033% CaCl_2_), and subjected to ultrasonic cleaning (3 × 3 min). Subsequently, specimens were fixed in a solution of 2.5% glutaraldehyde–2% paraformaldehyde, then stored at 4 °C for 24 h for later use.

### 2.2. Scanning Electron Microscopy (SEM)

Following the protocol of Bruno et al. [11], refrigerated experimental insect larvae of each instar were dehydrated through an ethanol gradient series (50%, 75%, 80%, 90%, 95%, and absolute ethanol), with each step lasting 30 min. The absolute ethanol dehydration step was repeated twice. After critical point drying (Leica EM CPD 300, Wetzlar, Germany), larval heads were dissected and mounted on specimen stages, positioned to expose dorsal, ventral, and lateral views. Samples were then gold-coated for 300 s using a sputter coater (Cressington 108auto, Watford, UK) and examined under a Sigma300 environmental scanning electron microscope (Zeiss, Jena, Germany) at an acceleration voltage of 10 kV for observation and imaging. Five to eight individuals from each larval instar were examined.

### 2.3. Transmission Electron Microscopy (TEM)

Heads of sixth- and seventh-instar larvae were dissected under a stereomicroscope (Zeiss Discovery V20, Jena, Germany) at 20× magnification and transferred to 0.65 mL centrifuge tubes (Axygen, Union City, CA, USA). These instars were selected based on BSF transition stage and consistent SEM-observed sensilla distribution across instars. The samples were rinsed with phosphate buffer for 30 min, fixed in 1% osmium tetroxide (4 °C, pH 7.4) for 2 h, and then washed with ddH_2_O to remove residual osmium tetroxide. Dehydration was carried out through a graded ethanol series (as described in the SEM protocol). The specimens were subsequently treated with propylene oxide for 10 min for solvent substitution, followed by infiltration in a 1:1 mixture of Epon 618 epoxy resin and acetone at room temperature for two days. The heads were then transferred to pure epoxy resin and polymerized at 60 °C for two additional days. To improve exposure of the antennae and mouthparts for sectioning, excess epoxy resin was carefully removed using a rasp. Ultrathin sections (50–70 nm) were prepared with an ultramicrotome (Leica EM UC7, Leica, Wetzlar, Germany), stained with uranyl acetate and lead citrate, and examined under a transmission electron microscope (JEM-1400Plus, JEOL, Tokyo, Japan) for imaging.

### 2.4. Nomenclature of Cephalic Sensilla

The classification and nomenclature of larval head sensilla were based primarily on external morphological features, including basal sockets and the presence and density of wall pores, following established systems for larval and dipteran sensilla as described by Barros et al. [10], Zacharuk and Shields [24], Solinas et al. [33], Courtney et al. [37], and Keil [38]. Internal ultrastructural features were described using the nomenclature of Solinas et al. [33], Keil [38], and Altner and Prillinger [39]. Sensillar subtypes were not further subdivided on the basis of length.

### 2.5. Quantitative Measurement and Analysis

The average count of each sensillum type was based on photomicrographs captured from dorsal, ventral, and lateral perspectives of the cephalic appendages. Morphometric parameters, including length, basal diameter, and socket dimensions, were measured for at least ten individuals per sensillum type per larval instar using ImageJ software (v1.53c; https://imagej.nih.gov/ij, accessed on 25 December 2025). All values are presented as mean ± standard deviation. Differences in measurements across instars were assessed by one-way ANOVA, followed by Tukey’s post hoc test for multiple comparisons. To evaluate whether sensillar growth conforms to Dyar’s rule [40,41], the mean size of each sensillum type was log-transformed (ln), and a linear regression was fitted against instar number. This yielded an exponential model (x = sensillum measurement in µm, y = instar) for estimating instar from key sensillum size. The growth ratio for each sensillum type was calculated as its size in a given instar divided by its size in the preceding instar; the final growth rate per structure represents the average of five consecutive ratios. All statistical computations were performed in R (v4.4.1; https://cran.r-project.org/, accessed on 25 December 2025).

## 3. Results

### 3.1. Structural Overview of the Cephalic Region in H. illucens Larvae

**Figure 1:**
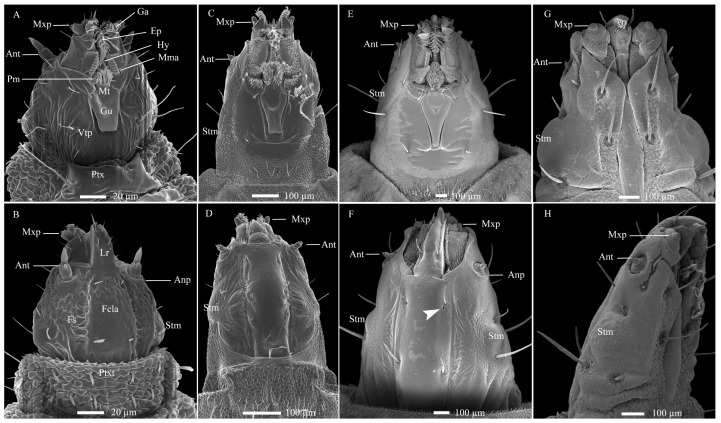
Cephalic structures and developmental changes in *Hermetia illucens* larvae from left to right across 1st, 3rd, 6th, and 7th instar. (**A**,**C**,**E**,**G**) Ventral views. (**B**,**D**,**F**) Dorsal views. (**H**) Lateral view. The white arrowhead indicates cuticular pores near the first pair of setae on the ventral view of the frontoclypeal apotome. Ant, antennae; Anp, antennal pad; Ep, epipharynx; Fs, frontal suture; Ga, galea; Gu, gula; Hy, hypopharynx; Lr, labrum; Mma, mandibular–maxillary apparatus; Mt, mentum; Mxp, maxillary palpus; Pm, prementum; Ptx, prothorax; Ptxt, prothorax tergum; Stm, stemma; Vtp, ventral plate of the head.

The larva of *H. illucens* is hemicephalic and apodal, with a prognathous, sclerotized head that is conical in shape (Figure 1). The head appears elongated, narrower than other body segments, and is retractile, connecting to the prothorax. Its coloration shifts from light to dark brown as the larvae matures (Figure S2). Distinct bright yellow markings are visible at the junctions of ventral sclerites and marginal membranes, especially in later instars. Ventrally, a large oral cavity dominates the anterior third of the head, framed by the genae and the ventral head plate. A gula is present between the postoccipital pits and the occipital foramen, bordered by dense cuticular processes (Figure 1A,C,E,F). Two semicircular membranous lobes extend toward the prementum posteriorly. The mandibular–maxillary complex is prominent, with a curved epipharynx medially connected to the hypopharynx. A membranous prementum is visible posteriorly, and all components are covered with cuticular processes (Figure S2). Dorsally, the convex frontoclypeal apotome lies between parallel frontal sutures, bearing one pair of sensilla chaetica at both ends (Figure 1B,D,F). Two symmetrical anterior genal lobes protect the mandibular–maxillary complex. The stemmata are medial, oval, dark brown, and covered by a thick chitinous layer, with one on each side (Figure 1 and Figure S2). The labrum is conical, robust, and projects forward with apical sensilla. SEM revealed cuticular pores near the first pair of setae on the frontoclypeal apotome, but not ventrally (Figure 1F), a diagnostic trait distinguishing *H. illucens* from other Stratiomyidae larvae [10,37].

### 3.2. Sensilla Types, Number, and Distribution on the Cephalic Appendages

Based on external morphology, wall pore presence/absence, pore distribution, socket flexibility, and dendritic structure, eight sensilla types were identified: sensilla basiconica (Sba), sensilla twig basiconica (Stb), sensilla campaniformia (Sca), sensilla placodea (Spl), sensilla ligulate (Sli), sensilla digitiformia (Sdi), sensilla trichodea (Str), and sensilla chaetica (Sch). Subtypes were further distinguished, including two for Sba, five for Stb, and three for Sch (Table 2). Cuticular pores were also observed on the frontoclypeal apotome (Figure 1F). Most types (Sba, Stb, Sca, Spl, and Sli) possess wall pores that allow chemicals to access the sensillar lymph cavity, supporting a primary chemosensory function, often with a secondary or possible mechanoreceptive role. These sensilla are concentrated at the antennae and palp apices. In contrast, Str and Sch lack wall pores but have flexible sockets, consistent with a purely mechanosensory role, and are sparsely distributed elsewhere. The finger-like Sdi on the maxillary palp also lacks pores but contains a single sensory neuron, suggesting a putative hygro-/thermoreceptive function. Critically, a consistent type-to-ultrastructure correlation was established, regardless of antennae side (left/right), palp identity, or developmental stage. This correlation is based on each type’s invariant positional arrangement on these appendages. The following section details each type’s morphology and ultrastructure.

#### 3.2.1. Sensilla on the Antenna

**Figure 2:**
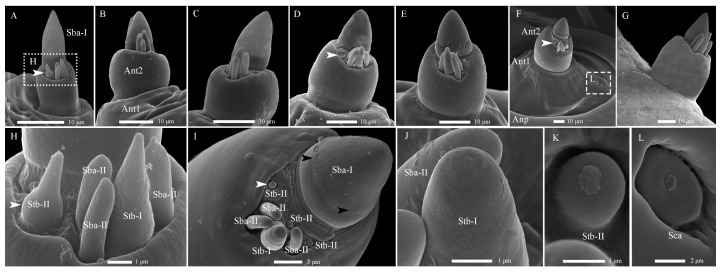
Antennal development and sensilla in *Hermetia illucens* larvae from left to right across the 1st to 7th instar. (**A**–**G**) Ventral views of the entire antenna across instars, with dotted and dashed box indicating the region magnified in following panel. (**H**,**I**) Morphology and sensilla distribution on the second antennal segment in the 1st and 6th instar, respectively. The white arrowhead marks the same sensillum type for comparing developmental changes between these stages. The black arrowheads indicate several large pores, each covered by an unidentified cone-shaped substance. (**J**–**L**) High-magnification images of sensilla types: sensilla basiconica and sensilla twig basiconica I (**J**), sensilla twig basiconica II (**K**), and sensilla campaniformia (**L**). Ant1-2, Antennae featuring two segments; Anp, antennal pad; Sba, sensilla basiconica; Stb, sensilla twig basiconica; Sca, sensilla campaniformia. Roman numerals denote sensilla subtypes.

In total, five types of antennal sensilla were identified on the two-segmented antennae. The antennae are inserted laterally and posterior to the joint of the mandible-maxillary complex on a large circular antennal pad. Among these, four types, including Sba-I, Sba-II, Stb-I, and Stb-II, are concentrated on the latero-dorsal view of the second antennal segment (Figure 1A,B and Figure 2A–I; Table 2). The remaining type, Sca, occurs on the proximal section of the first antennal segment, with one per antenna (Figure 2F).

##### Sensilla Basiconica (Sba)

These sensilla are conical in shape, featuring a rigid and stout base with blunt tips, each tightly encircled by a circular socket (Figure 2H,I; Table 2). Their cuticle presents two distinct types of pores: large pores located on the sidewall and tiny pores distributed across the smooth surface. Based on the presence and distribution of these cuticular pores, the Sba are further categorized into two subtypes: Sba-I and Sba-II.

**Figure 3:**
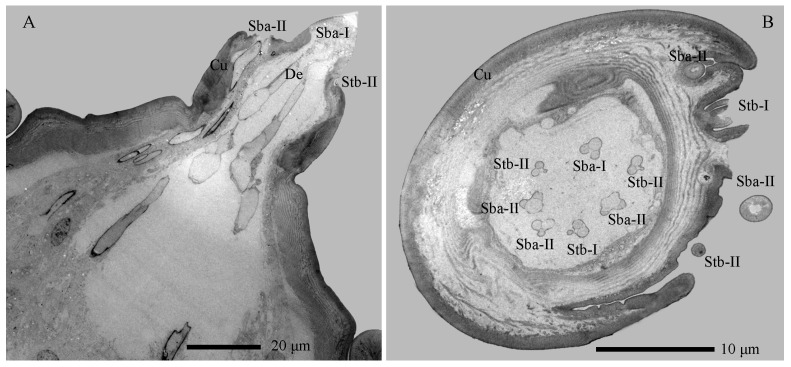
Antennal sections of *Hermetia illucens* larvae: longitudinal (**A**) and transverse (**B**) views showing sensilla. Cu, cuticle; Sba-I–II, sensilla basiconica I and II; Stb-I–II, sensilla twig basiconica I and II.

**Figure 4:**
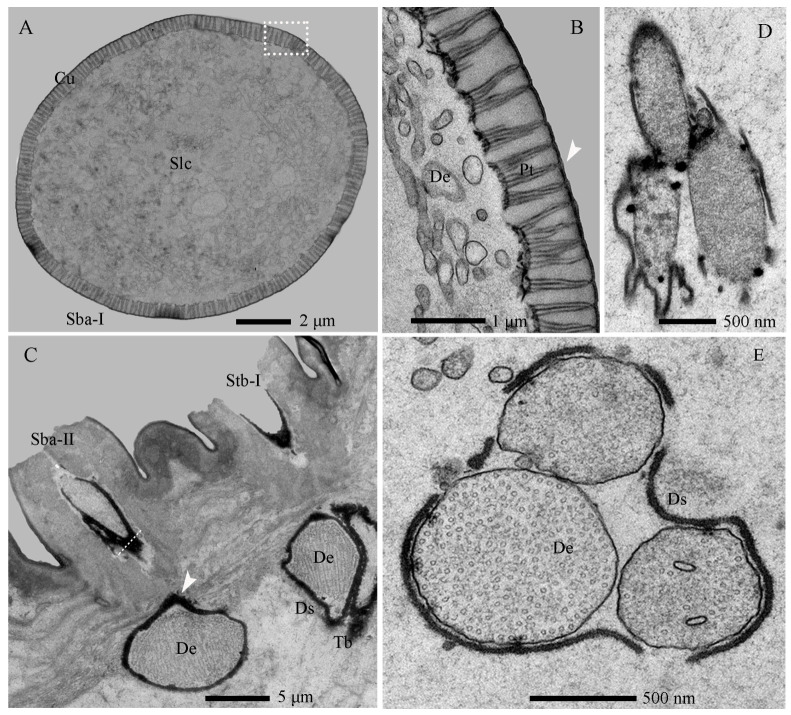
Internal structures of sensilla basiconica on the antennae of *Hermetia illucens* larvae. (**A**) Transverse section of a sensilla basiconicum I; (**B**) Higher magnification of the cuticle of a sensilla basiconicum I (white dotted box in panel (**A**)); (**C**) Longitudinal section of a sensilla basiconicum II, showing a dense dendritic sheath (arrowhead) surrounding the dendrites, with a sensilla twig basiconicum I visible to the right; The white dotted line marks the transverse section shown in (**D**); (**D**) Magnification of a sensilla basiconicum II (white solid line), showing three unbranched dendrites extending into the sensillar lymph cavity; (**E**) Innervation pattern of a sensilla basiconicum II in the cavity of the second antennal segment. Cu, cuticle; De, dendrites; Ds, dendritic sheath; Pt, pore tubule; Sba-I–II, sensilla basiconica I and II; Slc, sensillar lymph cavity; Stb-I–II, sensilla twig basiconica I and II; Tb, tubular body.

The Sba-I, a singular and prominent sensillum, is centrally positioned at the distal end of the antennal second segment (Figure 2A–G). It comprises a thick, smooth, non-porous annular base, contrasting with its grooved upper cone which bears numerous wall pores and sparse large pores (Figure 2I). Each large pore is covered by an unidentified cone-shaped substance, which was removed by KOH treatment (Figure S3). Ultrastructural analysis reveals the cone cuticle, approximately six times thinner than the base, is permeated by continuous tiny pores connected to electron-dense pore tubules (Figure 3A,B and Figure 4A,B). These tubules extend subcuticularly, closely associating with approximately 300 bundles of 6 dendritic branches with diameters between 0.1–0.4 µm (Figure 4B). These branches arise from 6 sensory neurons, forming a columnar receptor region beneath the cone.

The Sba-II, erect to slightly curved, are consistently found as a group of three on the inner distal surface of the second antennal segment (Figure 2A–I). They measure one-quarter to one-half the length of Sba-I across larval development, featuring a smooth, non-porous shaft with a porous apex (Figure 2J). Internally, TEM reveals three unbranched, ensheathed dendritic segments (0.6–0.9 µm in diameter) within a narrow lumen (Figure 4C). A deeper examination of the innervation pattern confirms that no numerical change occurs in these dendritic structures further proximally (Figure 4D,E).

##### Sensilla Twig Basiconica (Stb)

The Stb sensilla are short, straight, upright, and conical shapes with blunt tips (Figure 2H–K). Based on apical morphology, length, and location, they were categorized into Stb-I (Figure 2J) and Stb-II (Figure 2K) subtypes.

**Figure 5:**
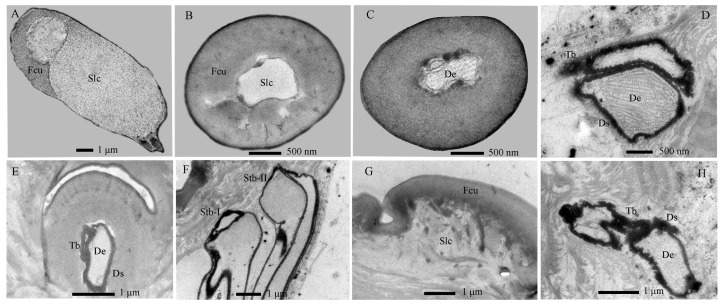
Internal structures of sensilla twig basiconica on the antennae of *Hermetia illucens* larvae. (**A**–**D**) Series of transverse sections through a sensillum twig basiconica I, progressing from the tip (**A**) to the mid-region (**B**,**C**) and basal portion (**D**). (**E**) Longitudinal section of a sensilla twig basiconicum II. (**F**) Dendrites of the two types of sensilla twig basiconica. (**G**) Longitudinal section of a sensilla campaniformium. (**H**) Dendrites of a sensilla campaniformium. Fcu, flexible cuticle; De, dendrites; Ds, dendritic sheath; Slc, sensillar lymph cavity; Stb-I–II, sensilla twig basiconica I and II; Tb, tubular body.

The Stb-I sensillum is the most prominent type in the lateral apical antennal field (Figure 2A–I). These fingerlike sensilla arise directly from the cuticle without a distinct socket and possess characteristically slit-like tips containing granular material (Figure 2J and Figure 5A). The distal one-third of the slender, poreless shaft encloses a flexible lumen, which contains a dendritic sheath within an empty cavity (Figure 5A,B). The cuticle increases in density toward the middle region, where unbranched dendrites begin to emerge (Figure 4C and Figure 5C,F). Immediately above the socket, the narrow lumen contains two ensheathed dendritic segments accompanied by a tubular body. Deeper innervation reveals no subsequent change in this dendritic pattern (Figure 3B, Figure 4C and Figure 5D).

The Stb-II are conical with smooth walls and a distinct papillate protrusion, each arising from a flexible circular socket (Figure 2I). A constant number of three is located on the inner side of the apical sensillum field and is relatively smaller than other types. Their morphology is spine-like in the first instar but remains stable from the second to seventh instars (Figure 2A–H). Ultrastructurally, a basal longitudinal section shows a tubular body and a large dendritic segment within the narrow lumen (Figure 5E). More basally, the lumen widens to enclose three dendritic segments, one of which consistently terminates as a tubular body attached to the socket’s flexible cuticle (Figure 3A,B and Figure 5F).

##### Sensilla Campaniformia (Sca)

The Sca sensillum is an ellipsoidal structure with a smooth surface and a distinct apical papilla, embedded within an oval cuticular depression (Figure 2F,L). It is singular, located exclusively on the inner base of the first antennal segment. Ultrastructurally, its flexible, nonporous shaft contains at least two dendritic segments, which terminate distally in a tubular body (Figure 5G,H).

#### 3.2.2. Sensilla on the Mouthparts

**Figure 6:**
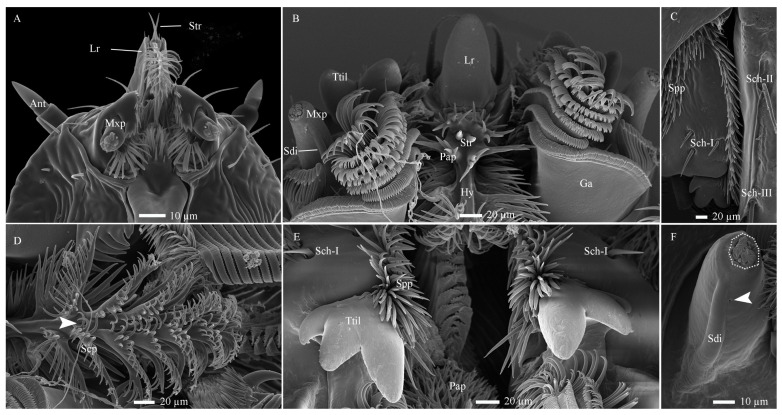
Distribution, morphology, and types of mouthpart sensilla in *Hermetia illucens* larvae. (**A**,**B**) Anterior region of the head appendages in the 1st and 6th instar larvae, respectively. (**C**,**D**) Ventral aspects of the mandible and epipharynx, respectively. (**E**) Dorsal aspects of the mandible. (**F**) Overview of the maxillary palp, with a dotted circle highlighting a cluster of sensilla at its tip. White arrowheads indicate cuticular pores at the corresponding structures. Ant, antennae; Ga, galea; Hy, hypopharynx; Lr, labrum; Mxp, maxillary palp; Pap, palmate processes; Scp, scaly processes; Spp, spiny processes; Sch-I–II, sensilla chaetica I–II; Sdi, sensilla digitiformia; Str, sensilla trichodea; Ttil, three-toothed incisor lobe.

**Figure 7:**
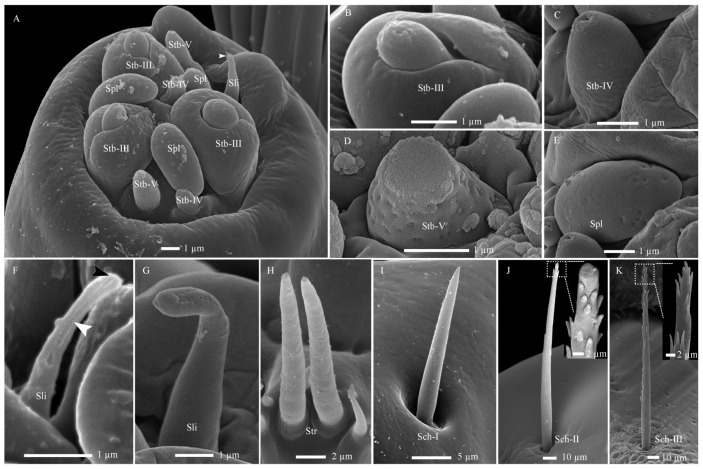
Fine morphology of mouthpart sensilla in *Hermetia illucens* larvae. (**A**) Sensilla cluster at the maxillary palp tip. The white arrowhead indicates a fragile region on the sensilla ligulate, predisposed to impairment at approximately one-third of its length. (**B**–**E**) High-magnification views of sensilla twig basiconica III, IV, V, and sensilla placodea, respectively. (**F**) Sensilla ligulate in the first-instar larva, showing a pore channel from the tip (black arrowhead) to the lower mid-region (white arrowhead), observed after unintentional damage to the sensillum. (**G**) An intact sensillum ligulatum in a sixth-instar larva. (**H**–**K**) Overall shapes of sensilla trichodea, sensilla chaetica I, II, and III, with insets revealing blunt and pointed saw-tooth gibbosities at their tips, respectively. Sli, sensilla ligulate; Spl, sensilla placodea; Stb-III, IV, V, sensilla twig basiconica III, IV, and V.

Ten distinct types of sensilla were identified on the mouthparts, including Str, Sch-I –III, Stb-III–V), Sli, Spl, and Sdi (Figure 6A–F and Figure 7A–K). Porous sensilla were concentrated at the maxillary palp tip, while non-porous types were sparsely distributed elsewhere (Table 2). Furthermore, the mouthpart surface exhibits several cuticular pores and cuticular processes. Based on morphological variations, these processes can be categorized into three subtypes: palmate, scaly, and spiny processes (Figure 6B–E).

##### Sensilla Twig Basiconica (Stb)

**Figure 8:**
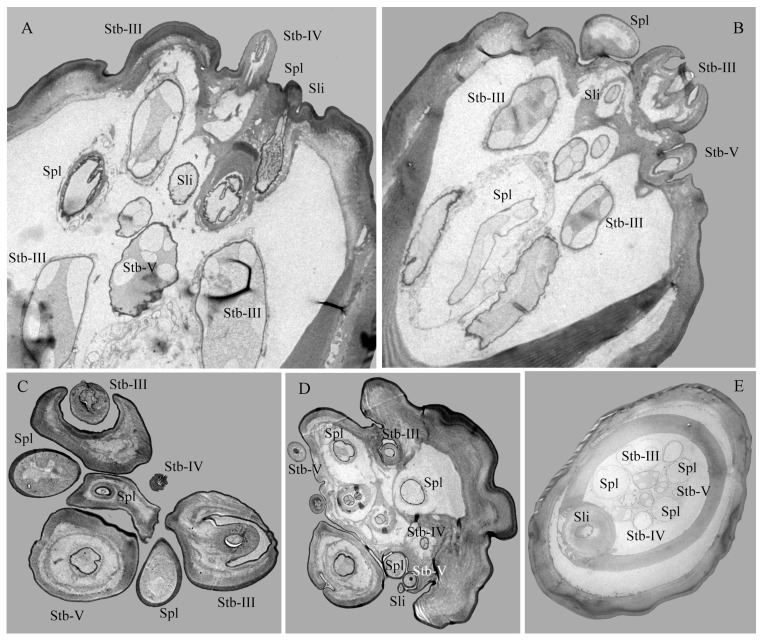
Sections of the maxillary palp of *Hermetia illucens* larvae at different regions: longitudinal (**A**,**B**) and transverse (**C**–**E**) views showing sensilla and internal structures. Stb-III–V, sensilla twig basiconica III, IV, and V; Sli, sensilla ligulate; Spl, sensilla placodea.

**Figure 9:**
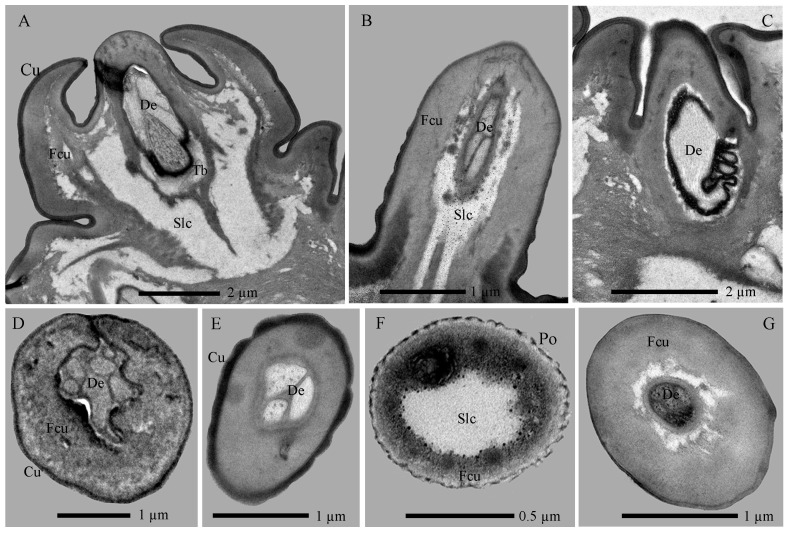
Internal structures of sensilla twig basiconica on the maxillary palp of *Hermetia illucens* larvae. (**A**–**C**) Longitudinal sections of sensilla twig basiconica III, IV, and V (left to right). (**D**–**G**) Transverse sections of sensilla twig basiconica III (**D**), IV (**E**), and V (**F**,**G**). Cu, cuticle; De, dendrites; Fcu, flexible cuticle; Po, pore; Slc, sensillar lymph cavity; Tb, tube body.

Stb-III possess a robust columnar base with a smooth wall and a conical apical protrusion featuring a single terminal pore (Figure 7A,B and Figure 8A–D). These sensilla are inserted directly into the cuticle at the apex of the palp, lacking a socket. The non-porous shaft is composed of a thick, flexible cuticle (Figure 9A). Apically, the sensillar lymph cavity encloses six to seven dendrites surrounded by a thin dendritic sheath (Figure 9D). More basally, the lumen widens and contains a distinct tubular body accompanied by six dendrites. Their somata reside at the palp base, while enveloping cell (trichogen and tormogen) projections extend distally.

Stb-IV are cylindrical sensory cones with smooth cuticular walls and a rounded apex featuring a distinct terminal pore (Figure 7A,C and Figure 8A,C). They are inserted directly into the membranous cuticle and are notably smaller than other sensillar subtypes. The terminal pore is surrounded by radial protrusions; unlike in other sensilla, the spaces between these protrusions contain electron-dense tubular structures (Figure 8C). Internally, a dendritic sheath attaches to a cuticular lamella within the lumen, and dendritic segments are located in the distal region of the sensillum. The basal segment is innervated by up to three dendrites and one tubular body (Figure 8A,B,E and Figure 9B,E).

Stb-V, the smallest and most conical type on the maxillary palp, are distributed in anterior–posterior pairs along the median axis near the apex margin (Figure 7A,D). Their surface is densely perforated by cuticular pores extending from base to tip. TEM analysis confirms the porous sensillar wall is embedded in flexible cuticle and encloses a hollow lumen (Figure 8A–D and Figure 9C,F). Median sections typically reveal three enlarged dendritic profiles giving rise to finer branches (Figure 9G). A single prominent tubular body, enclosed by a thick dendritic sheath, attaches to the cuticle. Deeper sections show three constant dendrites and a progressively condensed sheath (Figure 8E and Figure 9C).

##### Sensilla Placodea (Spl)

**Figure 10:**
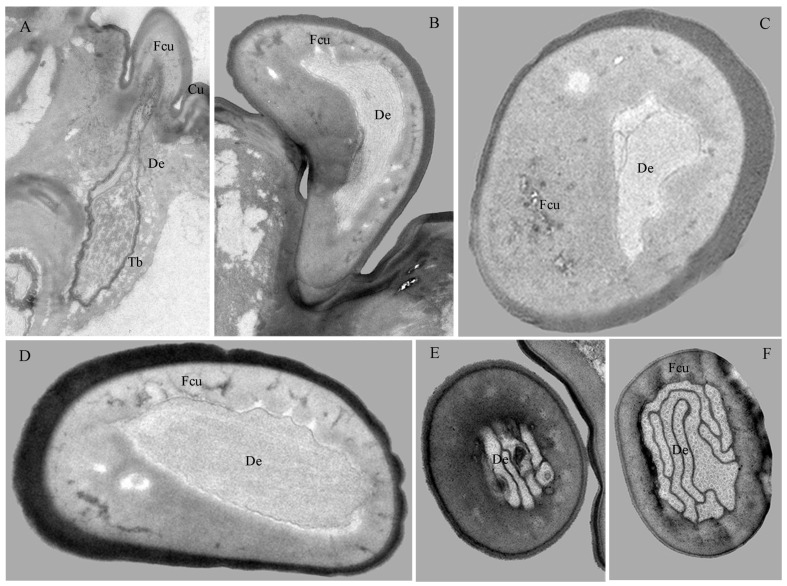
Internal structures of other sensilla on the maxillary palp of *Hermetia illucens* larvae. (**A**) Longitudinal sections of a sensilla ligulate. (**B**) Longitudinal sections of a sensilla placodeum. (**C**) Transverse sections of a sensilla ligulate. (**D**) Transverse sections of a sensilla placodeum. (**E**,**F**) Transverse sections of a sensilla digitiformium at the tip and mid-region, respectively. Cu, cuticle; De, dendrites; Fcu, flexible cuticle; Tb, tube body.

Spl are inwardly curved, sausage-shaped structures lying flat on the maxillary palp apex (Figure 7A,E). Each palp bears three sensilla, each positioned medially between Stb-III sensilla (Figure 7A and Figure 8C). The basal surface is smooth and non-porous, while the distal third bears numerous cuticular pores beneath a thin, electron-dense cuticular cover (Figure 7E, Figure 8C and Figure 10B,D). The sensillum is innervated by three sensory neurons whose dendrites, enveloped in a sheath, branch upon approaching an internal cavity. Within the cavity, they further divide into approximately 100 dendritic projections with vesiculated tips (Figure 8A–E and Figure 10B,D).

##### Sensilla Ligulate (Sli)

Sli are tongue-shaped structures located within a circular socket on the inner side of the maxillary apex, where they represent the longest sensillar type (Figure 7A,F,G). Each maxillary palp bears only one Sli. They taper gradually from the base, becoming lamellate at about two-thirds of their length. They exhibit a slightly twisted orientation parallel to the palp, with a broad, rough surface ending in a single apical pore (Figure 7F). Internally, two unbranched dendrites within a thin sheath traverse the lumen.

Basally, these converge into two unbranched dendrites and one tubular body, enclosed by a thick sheath (Figure 8E and Figure 10A,C).

##### Sensilla Digitiformia (Sdi)

The Sdi sensilla are conspicuous, finger-shaped structures that extend horizontally along the entire length of the maxillary palp (Figure 6F). Their cuticle is smooth and non-porous, featuring a subtle circular depression in the basal one-third and a distinct round cuticular pore located on the inner side of the apex. Each palp carries only one such sensillum. TEM reveals that the cuticle of the shaft has a spongy appearance and is delineated by the membrane of the lymph cavity. A single sensory neuron innervates the sensillum; its sheathed dendrite enters the cuticular channel to form a lamellar complex backed by a layer of microtubules that diminishes distally (Figure 10E,F).

##### Sensilla Trichodea (Str)

**Figure 11:**
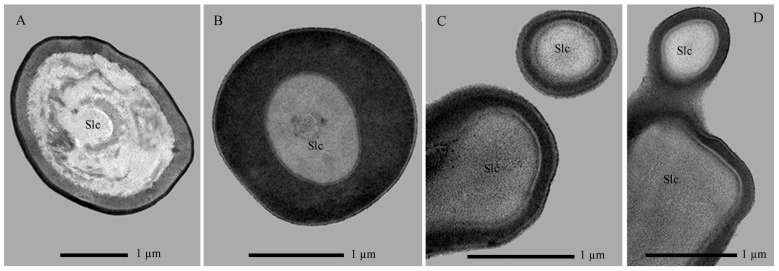
Transverse sections of mechanical sensilla on the mouthpart of *Hermetia illucens* larvae. (**A**) Sensilla trichodea. (**B**–**D**) Sensilla chaetica I, II, and III (left to right). Slc, sensillar lymph cavity.

Sensilla Str are hair-shaped structures, each situated within a slightly elevated, broad socket on the cuticle (Figure 6A,B). Characterized by a robust base, they taper gradually towards a finely pointed and slightly curved apex (Figure 7H). Their surface is smooth and entirely non-porous. These sensilla are exclusively located at the anterior end of the epipharynx, where they form a cluster of two units. TEM sections further reveal that the sensillar cuticle forms a thin, non-porous wall. Notably, the dendritic processes do not extend into or innervate this cuticular shaft structure (Figure 11A).

##### Sensilla Chaetica (Sch)

The Sch sensilla are bristle-shaped, with a distinct wide socket at the base where they connect to the cuticle. They taper gradually from the base to the tip, which is sharply pointed. Based on their morphology, they can be classified into three subtypes: Sch-I, Sch-II, and Sch-III (Figure 6C,E).

Sch-I possess a smooth surface and are situated within a concave, mortar-shaped socket. They incline at a 5–10° angle to the surface or curve basally to lie parallel to it (Figure 7I). Found exclusively at the mandibular base, two occur on the ventral side and three on the dorsal side of the molar lobe, totaling five sensilla. TEM reveals the sensillum has a thick, non-porous cuticular wall surrounding an inner lumen, which is not penetrated by dendritic branches (Figure 11B).

Both Sch-II and Sch-III are situated within circular sockets and share a similar overall bristle-like shape (Figure 6C). They are primarily distinguished by their surface texture: Sch-II is smooth with apical serrations only (Figure 7J), while Sch-III is densely serrated along its entire length (Figure 7K). These types are often intermixed in distribution across the labium, premental lobe, anterior frons-clypeus, and mandible-maxilla complex, with 1–3 sensilla per location. Internally, both possess a thick, non-porous cuticular wall and lack dendritic innervation (Figure 11C,D).

### 3.3. Changes in Antennal and Mouthpart Sensilla Across Instars of H. illucens Larvae

During larval ontogeny of *H. illucens*, the morphology, number, and distribution of cephalic sensilla (antennal and mouthpart) remain constant, with one exception (Figure 2A–G,I; Table 3). Stb-II exhibits a distinct morphology in the first instar, featuring a conical distal portion and a stout basal column, before transforming into the classic conical shape in subsequent instars. Statistical analysis of all sensilla types revealed significant differences in their dimensions (ANOVA, *p* < 0.001; Table 4). Furthermore, the calculated measures of central tendency and dispersion for cephalic and sensillar structures yielded exponential equations (Table 5). These equations indicate that growth in *H. illucens* follows a geometric progression, consistent with Dyar’s rule, allowing larval instar identification based on morphological measurements, particularly cephalic width. The average growth rate of chemoreceptors and hygro-/thermoreceptors is lower than that of mechanoreceptors (e.g., Str and Sch), when compared to cephalic width (Table 5).

## 4. Discussion

Insect cephalic sensilla, particularly those on the antennae and mouthparts, are functional traits that detect cues for habitat and food selection and trigger corresponding behaviors [31,38]. In response to similar ecological demands, these sensory structures may develop into specialized adaptations or convergent morphologies [24,29,33,42]. In this study, SEM and TEM analyses identified 15 distinct sensilla types on the antennae and mouthparts of BSF larvae, one Str, three Sch, two Sba, five Stb, one Sca, one Spl, one Sli, and one Sdi (Table 2), with only 2–9 sensilla per type. Of these, five types are located on the antennae (Figure 2H,I) and ten on the mouthparts (Figure 7A). Compared to adults of the same or related species [18,22,43,44], the larval sensilla exhibit relative simplicity in type, number, and distribution (Table 3 and Table 4). This simplicity reflects distinct sensory requirements between life stages. Adults, which interact with complex environments over long distances [20,21,22,23], possess diverse antennal sensilla, including uniporous grooved sensilla chaetica, coeloconic sensilla, porous trichoid sensilla, and porous complex sensilla [18,43], alongside maxillary palps bearing hundreds of sensory pits [44]. In contrast, larval sensory demands are constrained by their localized habitats and limited mobility [22,24,25]. This configuration aligns with patterns observed in larvae of related stratiomyids (e.g., *Neopachygaster maculicornis* (Hine, 1902) [45], *Stratiomys ruficornis* (Macquart, 1838) [46], *Ptecticus brevipennis* (Rondani, 1875) and *P. flavifemoratus* Rozkosny & Kovac, 1996 [47]) and concealed-dwelling Coleoptera (e.g., *Melolontha melolontha* (Linnaeus, 1758) [28], *Ophonus ardosiacus* (Lutshnik, 1922) [29], and *Anoplophora glabripennis* (Motschulsky, 1853) [30]) whereas Dipteran larvae with differing life histories (e.g., *Chrysomya megacephala* (Fabricius, 1794) [22], and *Aedes albopictus* (Skuse, 1894) [48]) display distinct sensory arrangements. Based on their ultrastructural features, these sensilla are inferred to function primarily as chemoreceptors involved in olfaction, mechanosensation, and hygro-/thermoreception during food and habitat evaluation [24,25,27,39]. Furthermore, these chemosensory organs, including olfactory (Sba, Spl) and tactile (Stb, Sca, Sli) sensilla, are concentrated at the tips of the antennae and maxillary palps. However, their low number, restricted distribution, and small surface area relative to adults indicate a limited olfactory perception capacity [18,44]. This pattern reflects the constrained life history and short-range chemosensory ecology of larvae [22,24]. Notably, significant interspecific heterogeneity exists, with antennal length serving as a key distinguishing feature among ecological–behavioral groups and reflecting variation in olfactory capacity, as reported for larval groups of Cecidomyiidae [33].

Interesting, this study presents a significant revision of larval antennal segmentation in *H. illucens*, challenging conventional morphological interpretations. Through SEM and TEM analysis, we identified a previously overlooked proximal segment originating from a deep, oval antennal pad (Figure 2F). This retractile structure has frequently been omitted in prior descriptions relying on traditional methodologies [11,35,37]. While the resulting two-segmented appearance aligns with the general configuration observed in Brachycera, including Stratiomyidae, Pelecorhynchidae, Athericidae, and certain Bombyliidae (e.g., *Heterotropus* and *Glabellula*) [37,49], our findings fundamentally diverge from traditional segmentation schemes regarding homology. Specifically, we demonstrate that the distalmost structure, historically classified as the terminal antennal segment, lacks a definitive articulation with the adjacent segment. Based on this key morphological criterion, we reclassify this structure not as a true segment, but as a Sba. Consequently, we propose a revised model for antennal segmentation in Stratiomyidae larvae. This corrected division not only provides an accurate anatomical framework but may also facilitate further investigations into the ecological adaptations of dipteran larval structures [33,35,37,44].

### 4.1. Correlating Morphology with Putative Function in the Sensilla of H. illucens Larvae

Cuticular porous sensilla, characterized by wall and/or terminal pores, mainly serve olfactory and gustatory functions [39]. Their porous structure permits both volatile and contact chemicals to enter the lymph cavity and bind to corresponding receptors; certain morphological subtypes may additionally serve a mechanosensory role when associated with a tubular body [23,28]. In this study, we identified ten pore-bearing sensillum types, including Sba, Spl, Stb, Sca, Sli and their respective subtypes, which are predominantly clustered at the antennal and maxillary palp tips (Figure 2A,I and Figure 6A). The presence of multiple neurons (≥2) within these sensilla further supports their chemosensory specialization (Table 2; Figure 3A,B and Figure 8A–E). Based on pore distribution and ultrastructure, multiporous sensilla (Sba-I, Sba-II, Spl) are interpreted as olfactory organs responsive to volatile compounds [32,39,42,50,51]. Furthermore, sensillum Sba-I exhibits several large pores on its surface, each containing an unidentified cone-shaped substance (Figure 2I and Figure S3). We propose that this material may be secreted through the pores, suggesting a potential gustatory function for this sensillum [27,38,39]. This morphology is analogous to the large pores observed on the maxillary palps of *Helicoverpa armigera* (Hübner, 1809) caterpillars (peg sensillum no. 4) [52] and *A. glabripennis* beetle larvae (sensilla basiconica type 1) [30] following KOH treatment. Nevertheless, further investigations employing similar or electrophysiological techniques are necessary to confirm the specific function of these structures. In contrast, sensilla with terminal pores (Stb-I–V, Sca, Sli) are classified as contact chemoreceptors involved in gustation. The occurrence of a tubular body in several of these subtypes implies a bimodal function, combining gustatory with mechanosensory capabilities [22,39,44,48]. The functional significance of these cephalic chemosensilla is further supported by the observed behavior of larvae actively orienting toward fresh food, a finding analogous to chemically guided foraging in the root-feeding beetle *M. melolontha* [28].

In contrast to porous sensilla, nonporous sensilla enable insects to perceive a range of physical stimuli, including touch, air movement, sound, gravity, humidity, temperature, and pressure, as well as chemical stimuli such as CO_2_ [39,53]. In *H. illucens* larvae, five nonporous types were identified, Str and Sch-I-III, as well as Sdi, distributed across different mouthpart regions (Figure 6A–F). Based on dendritic presence within the sensillar lymph cavity, these can be divided into two groups: Str and Sch, which lack dendrites, and Sdi, which contains a single dendrite. The paired Str sensilla project erectly from the epipharyngeal surface, where their inwardly curved tips, anchored in a tight socket, likely monitor semi-liquid food consistency during feeding and trigger timely sensory feedback [22,39,44,48,53]. Sch-I is exclusively located on the mandibular surfaces, with morphology and placement consistent with mechanoreceptors that monitor mandibular position and movement [17,54]. Sch-II and Sch-III are scattered over other mouthpart components and may act as proprioceptors or protective mechanoreceptors, mitigating mechanical stress during feeding [22,48,53,55].

The Sdi has been documented on the maxillary and/or labial palps of holometabolous insects within Coleoptera and Lepidoptera (in both adults and larvae), such as *M. melolontha* [28], *Ctenicera destructor* (Brown, 1950) [56], *A. glabripennis* [30], *Tomicus yunnanensis* Kirkendall & Faccoli, 2008 [54], and *H. armigera* [52]. In contrast, it is rarely reported in Diptera [10,22,32,33,35,44,47,48]. Functionally, this sensillum type remains ambiguous. Some studies describe lamellated dendrites within its lymph cavity, a morphology typical of hygro-/thermo- or CO_2_ receptors [52,57]. Others, however, report the absence of such structures and instead provide electrophysiological evidence for sensitivity to mechanical vibration, classifying the Sdi as a vibroreceptor [56]. In *H. illucens* larvae, a single Sdi is present on each maxillary palp. Thermo-hygrosensory perception is critical for larval survival, and the maxilla actively assists during feeding. Our ultrastructural analysis revealed highly lamellated dendritic structures within this sensillum, a feature distinctly different from that reported in *H. armigera* by Keil [52] but characteristic of thermo-hygroreceptors [57]. Consequently, we propose that the digitiform sensillum in *H. illucens* larvae functions primarily as a hygro-/thermoreceptive organ.

### 4.2. Ontogenetic Changes in the Sensilla of H. illucens Larvae

Throughout the larval development from the 1st to the 7th instar, cephalic width and sensilla dimensions serve as reliable morphometric indicators for instar determination (Table 5). Furthermore, the larval morphology of *H. illucens* remains largely consistent across different instars, a characteristic shared with other Stratiomyidae. This morphological uniformity implies that investigating the larval sensory biology of related Stratiomyidae species using only one or two instar stages is sufficient [10,36,55]. The antennal and mouthpart sensilla exhibit a stable repertoire in typology, number, and distribution, with only minor variations such as in Stb-II being negligible (Figure 2H). Among sensilla types, mechanosensilla show greater robustness as developmental markers than chemosensilla, likely due to their more rigid structure and consistent growth patterns. The size of each sensillum type increases exponentially during growth, a pattern also documented in other Diptera and Coleoptera larvae with comparable life histories [30,33]. This pattern reflects ecological adaptation. For instance, in Cecidomyiidae larvae, olfactory organ size varies among ecological–behavioral groups, correlating with the specific larval habitats selected by adults [33]. Similarly, among carabid beetles, surface-active walkers and soil diggers possess more mouthpart mechanosensilla, whereas larvae inhabiting confined substrates like litter or soil interstices are enriched in contact chemosensilla [29]. While insect organ growth generally provides increased space for sensilla, promoting their expansion in size and number [11,33,38], larvae developing in stable microhabitats, such as gall-makers *Allocontarina sorghicola* (Solinas, 1986) [33] or trunk-borers *T. yunnanensis* [54], show no significant increase in sensilla number, likely due to buffered environmental conditions. This adaptive reduction is further evidenced by the simpler and fewer cephalic sensilla in larvae compared to adults. Notably, in *H. illucens* larvae, chemical sensilla are fewer in type and less abundant on the antennae than on the mouthparts. This distribution suggests that mouthpart sensilla play a more critical role in feeding and microhabitat selection within semi-liquid substrates, positioning the mouthparts as the predominant sensory organs during this life stage. The significantly reduced size of larval antennae compared to adults may correspond to a lesser reliance on long-range olfaction [18,44]. Furthermore, three types of cuticular processes, including palmate, scaly, and spiny, are densely arranged around the anterior hypopharyngeal opening. Analogous structures in other terrestrial Stratiomyidae imply a function in food filtration during feeding [11,37]. These processes disappear in the 7th instar (Figure 1G,H), coinciding with the cessation of feeding, gut clearance, and reduced mobility. Consequently, future research should focus on electrophysiological responses and key chemosensory genes (e.g., OBPs, CSPs, Ors, SNMP, IR) in *H. illucens* larvae, particularly during the final phase of the sixth instar or the seventh instar. Deciphering the underlying mechanisms of olfaction and gustation at these stages is essential for optimizing rearing systems via behavioral manipulation [19,58].

## 5. Conclusions

This ultrastructural study provides the first detailed description of the sensory organs on the cephalic appendages of *H. illucens* larvae, an insect of global importance for waste bioconversion and sustainable resource production. A total of 15 distinct sensillum types were characterized and found to remain constant in number and position across larval development. Antennal sensilla comprise five types: sensilla basiconica I and II (Sba-I, II) and sensilla twig basiconica I and II (Stb-I, II), along with sensilla campaniformia (Sca). Present in sets of 1–3 units at the antennal apex, these sensilla are characterized by cuticular pores and are innervated by 2–6 sensory neurons, suggesting chemosensory functions, primarily olfaction and gustation, with possible additional mechanosensory roles. The mouthparts host ten sensillum types, sensilla twig basiconica III–V (Stb-III–V), sensilla placodea (Spl), sensilla ligulata (Sli), sensilla digitiformia (Sdi), sensilla trichodea (Str), and sensilla chaetica I–III (Sch-I–III), each occurring in groups of 2–8 units. Stb-III–V, Spl, and Sli are localized exclusively at the tips of the maxillary palps, while Sdi is situated laterally on the palps. The remaining sensilla, Str and Sch, are sparsely distributed across other mouthpart segments. Functionally, the terminal porous sensilla (Stb-III–V, Spl, Sli), innervated by 2–6 neurons, are interpreted as bimodal chemo- and/or mechanoreceptors. In contrast, Sdi exhibits a non-porous cuticle and a single sensory cell, indicative of a thermo-/hygrosensory role. Non-porous sensilla Str and Sch, which lack dendrites, are identified as mechanoreceptors. These findings establish a morphological and functional framework for larval sensation, offering a scientific basis for future chemosensory mechanisms and optimize rearing system through behavioral modulation.

## Figures and Tables

**Table 1:** Criteria for larval instar determination in *Hermetia illucens*.

Larval Instars	Cephalic Width (µm)	Body Color	Duration (Days)
1	111.09 ± 5.43	whitish	0.1–0.2
2	195.52 ± 32.96	cream white	0.3–0.4
3	373.97 ± 15.12	Light yellow	3–5
4	544.63 ± 19.70	Dark yellow	3–5
5	906.73 ± 28.27	Yellowish-brown	3–5
6	1321.13 ± 115.00	brown	3–5
7	796.55 ± 38.63	dark brown	7–10

**Table 2:** Hypothesized function, external morphology, distribution, and dendritic structure of sensilla on the antennae and mouthparts of *Hermetia illucens* larvae.

Hypothesized Function	Sensillum	Shape	Location	Pores	Socket	Surface	Dendrites
Olfaction	Sba-I	Large, robust, straight cone with a blunt tip	Ant	Wp	Tg	smooth	6 Bds, ca. 300 bundles
	Sba-II	Small, slender, straight cone with a blunt tip	Ant	Tp	Tg	smooth	3 Uds, No Tb
	Spl	Large, incurved, sausage-shaped protrusion	Mtp	Wp	Ub	smooth	3 Bds, ca. 100 bundles
Tactile and mechanoreceptive function	Stb-I	Large, straight, and robust cone with a blunt, cracked tip	Ant	Tp	Ub	smooth	2 Uds, 1 with Tb
	Stb-II	Small, straight, cone with a papillate tip	Ant	Tp	Wd	smooth	2 Uds, 1 with Tb
	Stb-III	Large, robust, straight, column with a conical apical protrusion	Mtp	Tp	Ub	smooth	5–6 Uds, 1 with Tb
	Stb-IV	Small, straight, cylindrical cone with a terminal apical pit	Mtp	Tp	Ub	smooth	3 Uds, 1 with Tb
	Stb-V	Small, straight, cylindrical cone with an obtuse, coarse tip	Mtp	Wp	Ub	smooth	4 Uds, 1 with Tb
Tactile function	Sca	Oval, depressed cap with a central papillate protrusion	Ant	Tp	Wd	smooth	1 Uds, 1 with Tb
	Sli	Thin hair with a tongue-shaped tip	Mtp	Tp	Wd	smooth	1 Uds, 1 with Tb
Hygro-/thermorception	Sdi	Finger-shaped protrusion in an elongated cuticular recess	Mtp	Ap	Ub	smooth	1 Uds, No Tb
Mechanoreception	Str	Thin, straight hair	Mtp	Ap	Tg	smooth	Tb not observed
	Sch-I	Straight, rigid, shape bristle	Mtp	Ap	Wd	smooth	Tb not observed
	Sch-II	Straight, slender bristle with an obtuse tip	Mtp	Ap	Wd	serrated	Tb not observed
	Sch-III	Straight, slender bristle with a serrated tip	Mtp	Ap	Wd	serrated	Tb not observed

Ant, antennae; Ap, Aporous; Bds, branched dendrites; Mtp, mouthpart; Sba, sensilla basiconica; Sca, sensilla campaniformia; Sch, sensilla chaetica; Sdi, sensilla digitiformia; Sli, sensilla ligulate; Spl, sensilla placodea; Str, sensilla trichodea; Stb, sensilla twig basiconica; Tb, tubular body; Tg, tight; Tp, terminal pore; Uds, unbranched dendrites; Wd, wide; Wp, wall pore; Ub, Unobvious; Roman numerals denote sensilla subtypes.

**Table 3:** Abundance of antennal and mouthpart sensilla during larval ontogeny in *Hermetia illucens*.

Type	Larval Stages						
	1	2	3	4	5	6	7
Sba-I	2	2	2	2	2	2	2
Sba-II	6	6	6	6	6	6	6
Stb-I	2	2	2	2	2	2	2
Stb-II	6	6	6	6	6	6	6
Stb-III	6	6	6	6	6	6	6
Stb-IV	4	4	4	4	4	4	4
Stb-V	4	4	4	4	4	4	4
Sca	2	2	2	2	2	2	2
Spl	2	2	2	2	2	2	2
Sli	2	2	2	2	2	2	2
Sdi	2	2	2	2	2	2	2
Str	2	2	2	2	2	2	2
Sch-I	6	6	6	6	6	6	6
Sch-II	6	6	6	6	6	6	6
Sch-III	9	9	9	9	9	9	9

Sba, sensilla basiconica; Sca, sensilla campaniformia; Sch, sensilla chaetica; Sdi, sensilla digitiformia; Sli, sensilla ligulate; Spl, sensilla placodea; Str, sensilla trichodea; Stb, sensilla twig basiconica. Roman numerals denote sensilla subtypes. All abbreviations are consistent with those used in the subsequent tables.

**Table 4:** Sensillar length on the antennae and mouthparts during larval ontogeny in *Hermetia illucens*.

Type	Larval Stages (µm)						
	1	2	3	4	5	6	7
Sba-I	11.2 ± 0.4	12.7 ± 0.1	15.9 ± 0.2	16.1 ± 0.6	20.7 ± 0.1	21.2 ± 0.3	23.0 ± 0.1
Sba-II	3.49 ± 0.11	5.29 ± 0.24	5.69 ± 0.53	7.74 ± 0.42	7.76 ± 0.18	8.9 ± 0.27	10.76 ± 0.33
Stb-I	3.9 ± 0.5	5. 5 ± 0.1	8.8 ± 0.1	12.2 ± 2.9	13.4 ± 2.1	17.4 ± 0.8	18.0 ± 0.7
Stb-II	1.0 ± 0.2	1.2 ± 0.1	1.5 ± 0.3	1.8 ± 0.8	1.9 ± 0.2	2.2 ± 0.3	2.4 ± 0.3
Stb-III	2.5 ± 0.2	2.8 ± 0.1	3.9 ± 0.1	4.7 ± 0.1	5.6 ± 0.1	6.1 ± 0.1	6. 3 ± 0.2
Stb-IV	1.4 ± 0.1	1.6 ± 0.4	2.4 ± 0.1	2.8 ± 0.5	3.0 ± 0.2	3.7 ± 0.1	4.1 ± 0.3
Stb-V	1.1 ± 0.1	1.2 ± 0.1	1.4 ± 0.1	1.6 ± 0.2	1.6 ± 0.4	1.8 ± 0.1	1.8 ± 0.4
Sca	0.2 ± 0.0	0.2 ± 0.1	0.3 ± 0.0	0.3 ± 0.0	0.3 ± 0.0	0.5 ± 0.01	0.6 ± 0.0
Spl	1.3 ± 0.2	2.2 ± 0.2	2.4 ± 0.1	2.8 ± 0.2	3.4 ± 0.3	4.0 ± 0.6	4.4 ± 0.6
Sli	2.6 ± 0.1	3.2 ± 0.1	3.6 ± 0.0	3. 9 ± 0.1	4.1 ± 0.1	4.6 ± 0.2	4.8 ± 0.1
Sdi	9.3 ± 0.6	12.5 ± 0.5	17.1 ± 1.7	26.3 ± 1.7	32.9 ± 2.9	43.35 ± 2.7	47.9 ± 1.4
Str	4.2 ± 0.1	8.1 ± 0.1	14.9 ± 0.7	19.1 ± 0.1	29.7 ± 0.3	45.5 ± 9.7	69.6 ± 12.2
Sch-I	5.6 ± 1.5	8.8 ± 3.1	18.8 ± 7.8	22.2 ± 8.9	28.4 ± 12.2	57.7 ± 11.3	83.2 ± 13.2
Sch-II	5.9 ± 0.8	7.7 ± 0.2	9.6 ± 2.8	28.4 ± 4.9	30.9 ± 4.6	50.0 ± 18.3	52.8 ± 19.1
Sch-III	7.5 ± 0.1	9.2 ± 1.1	12.9 ± 2.3	23.5 ± 2.1	28.5 ± 2.2	72.4 ± 22.4	96.2 ± 3.8

**Table 5:** Modeling the exponential growth and mean growth rate of the cephalic width and its sensilla during larval ontogeny in *Hermetia illucens*.

Measure	Equation y = ex	r^2^	Average Rate of Growth
Cephalic width	y = 73.598e^0.4959x^	0.9914	1.65
Sba-I	y = 9.8928e^0.1344x^	0.9469	1.18
Stb-I	y = 3.1621e^0.2992x^	0.9547	1.36
Stb-III	y = 2.0632e^0.1924x^	0.9694	1.20
Spl	y = 1.2945e^0.1867x^	0.9578	1.28
Sdi	y = 7.3107e^0.288x^	0.9607	1.36
Str	y = 3.1736e^0.4482x^	0.9974	1.21
Sch-I	y = 3.8627e^0.4388x^	0.9844	1.62
Sch-III	y = 3.9395e^0.4483x^	0.9679	1.64

## Data Availability

The original contributions presented in this study are included in the article/Supplementary Materials. Further inquiries can be directed to the corresponding authors.

## Supplementary Materials

The following supporting information can be downloaded at: https://www.mdpi.com/article/10.3390/insects17040362/s1, Figure S1: Morphological changes in *H. illucens* larvae from the left to right across the first to seventh instars. Figure S2: Stereomicroscope images of cephalic structures and developmental changes in *H. illucens* larvae from the 3rd to 7th instars, shown in ventral (top) and dorsal (bottom) views. Figure S3: Sensilla basiconica I on the fifth instar larva following KOH treatment, showing several large, sunken pores (arrowhead) on the damaged cuticular surface.
