# Supplementary material for: Ontogenetic Variations in the Sensory Organ Structure and Morphology on the Cephalic Appendages of Hermetia illucens (Diptera: Stratiomyidae) Larvae
Source: Insects. 2026 Mar 25;17(4):362. doi: 10.3390/insects17040362 (PMC13115912; doi:10.3390/insects17040362)
Supplement: Supplementary file 1 [file insects-17-00362-s001.zip › insects-4176084-supplementary.pdf]

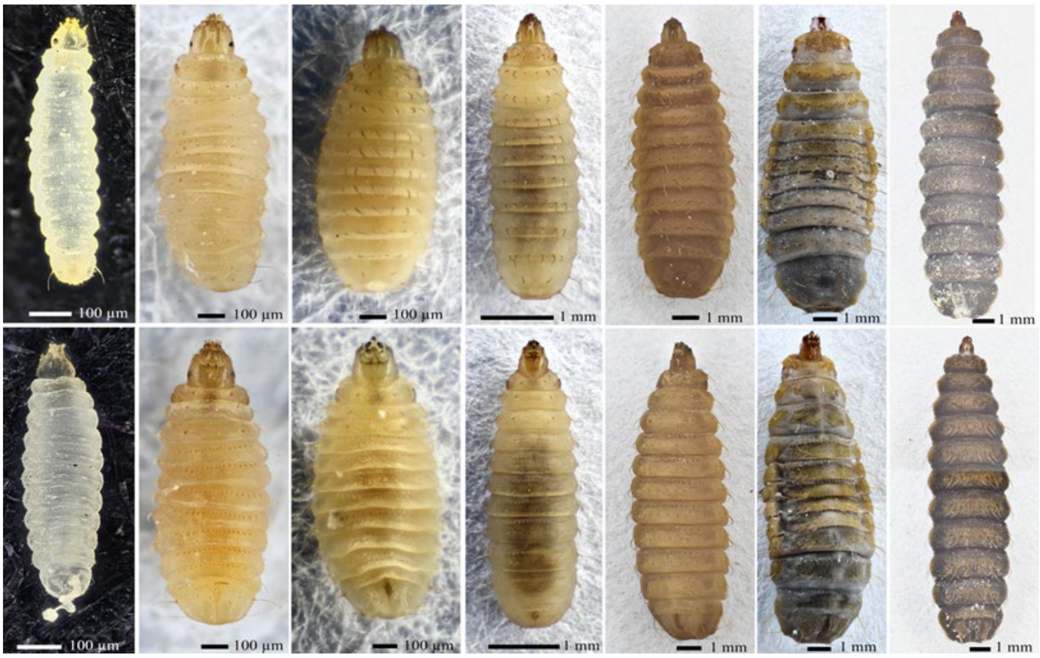

Figure S1. Morphological changes in *Hermetica illucens* larvae from the left to right across the first to seventh instars. Dorsal (top) and ventral (bottom) views are shown.

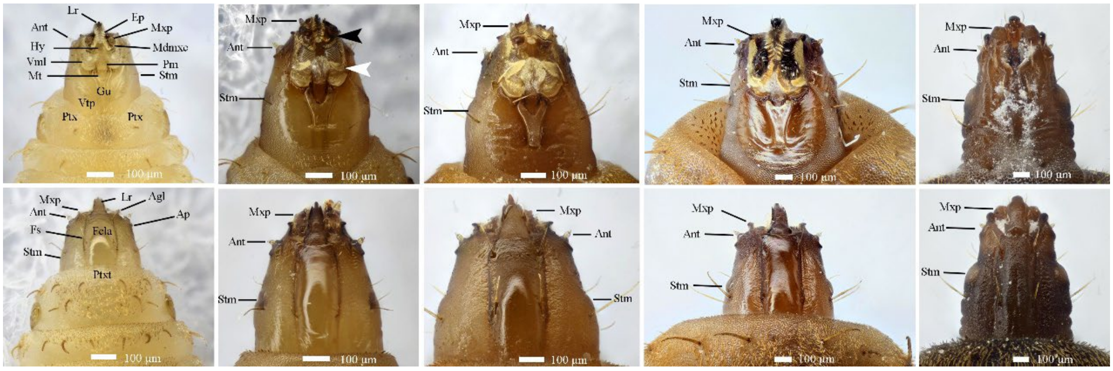

Figure S2. Stereomicroscope images of cephalic structures and developmental changes in *H. illucens* larvae from the 3rd to 7th instars, shown in ventral (top) and dorsal (bottom) views. The white and black arrowheads indicate the bright yellow membranous structure and the mandibular-maxillary apparatus, respectively. Abbreviations: Ant, antennae; Ep, epipharynx; Fs, frontal suture; Gu, gula; Hy, hypopharynx; Lr, labrum; Mdmxc, mandibular-maxillary apparatus; Mt, mentum; Mxp, maxillary palpus; Pm, prementum; Ptx, prothorax; Ptxt, prothorax tergum; Stm, Stemma; Vml, ventral membranous lobe; Vtp, ventral plate of the head.

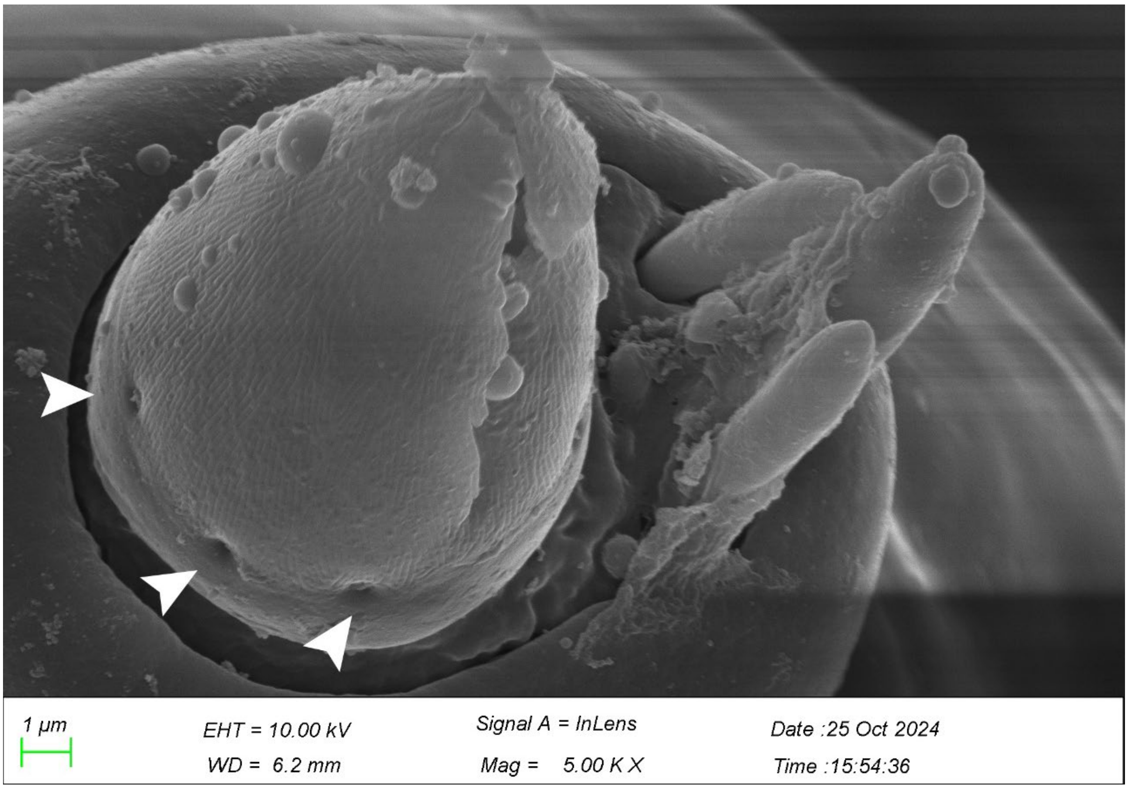

Figure S3. Sensilla basiconica I on the fifth instar larva following KOH treatment, showing several large, sunken pores (arrowhead) on the damaged cuticular surface.
